# Stability of extemporaneously compounded amiloride nasal spray

**DOI:** 10.1371/journal.pone.0232435

**Published:** 2020-07-10

**Authors:** Venkata Yellepeddi, Casey Sayre, Anna Burrows, Kevin Watt, Simon Davies, John Strauss, Marco Battaglia

**Affiliations:** 1 Division of Clinical Pharmacology, Department of Pediatrics, School of Medicine, University of Utah, Salt Lake City, Utah, United States of America; 2 Department of Pharmaceutics and Pharmaceutical Chemistry, College of Pharmacy, University of Utah, Salt Lake City, Utah, United States of America; 3 College of Pharmacy, Roseman University of Health Sciences, South Jordan, Utah, United States of America; 4 Centre for Addiction and Mental Health, Toronto, Ontario, Canada; 5 Department of Psychiatry, University of Toronto, Toronto, Ontario, Canada; University of Porto, PORTUGAL

## Abstract

Anxiety disorders (AD) are the most common mental conditions affecting an estimated 40 million adults in the United States. Amiloride, a diuretic agent, has shown efficacy in reducing anxious responses in preclinical models by inhibiting the acid-sensing ion channels (ASIC). By delivering amiloride via nasal route, rapid onset of action can be achieved due to direct “nose-to-brain” access. Therefore, this study reports the formulation, physical, chemical, and microbiological stability of an extemporaneously prepared amiloride 2 mg/mL nasal spray. The amiloride nasal spray was prepared by adding 100 mg of amiloride hydrochloride to 50 mL of sterile water for injection in a sterile reagent bottle. A stability-indicating high-performance liquid chromatography (HPLC) method was developed and validated. Forced-degradation studies were performed to confirm the ability of the HPLC method to identify the degradation products from amiloride distinctively. The physical stability of the amiloride nasal spray was assessed by pH, clarity, and viscosity assessments. For chemical stability studies, samples of nasal sprays stored at room temperature were collected at time-points 0, 3 hr., 24 hr., and 7 days and were assayed in triplicate using the stability-indicating HPLC method. Microbiological stability of the nasal spray solution was evaluated for up to 7 days based on the sterility test outlined in United States Pharmacopoeia (USP) chapter 71. The stability-indicating HPLC method identified the degradation products of amiloride without interference from amiloride. All tested solutions retained over 90% of the initial amiloride concentration for the 7-day study period. There were no changes in color, pH, and viscosity in any sample. The nasal spray solutions were sterile for up to 7 days in all samples tested. An extemporaneously prepared nasal spray solution of amiloride hydrochloride (2 mg/mL) was physically, chemically, and microbiologically stable for 7 days when stored at room temperature.

## Introduction

Anxiety disorders (AD) are the most common mental illnesses in every age group, affecting 25% of children and an estimated 40 million adults in the United States [1]. Risk factors for developing AD include genetics, life adversities, and subtle brain chemistry alterations [1].

Pharmacological and cognitive-behavioral interventions, alone or in combination, are typically employed to treat AD [2, 3]. Contemporary first-line pharmacological agents for AD include selective serotonin reuptake inhibitors (SSRIs) and some serotonin noradrenaline-reuptake inhibitors (SNRIs). SSRIs and SNRIs are better tolerated than older generation tricyclic antidepressants and show moderate-to-good effectiveness. However, the effectiveness and duration of treatment are not significantly different compared with tricyclic antidepressants, and many people experience relapse [4]. This highlights the unmet need for improved therapeutics in AD [2, 3].

In a series of comparative human and preclinical studies of responses to CO_2_ (an unconditioned stimulus that evokes panic-like responses in humans at risk for panic disorder and hyperventilation in rodents) [5–10], we reported that life adversities enhance the liability to anxiety and pain through the enrichment of acid-sensing ion channel (ASIC) genes -1 and -2 [11–13]. Coherent with these findings, ASIC-antagonist amiloride normalizes the enhanced anxious and nociceptive responses that are proper of mice exposed to early disrupted maternal care and the nociceptive responses of rats that underwent prenatal maternal stress [12, 14]. In a series of randomized human clinical trials, amiloride was shown to be safe and effective in humans for the treatment of cystic fibrosis following inhalational administration [15–18]. Furthermore, amiloride inhalation at 10 mg dose showed minimal systemic absorption in adolescent and adult cystic fibrosis patients when compared with oral dosing [17]. Taken together, these data suggest that amiloride has the potential to provide safe and effective treatment in humans with certain AD, and for some conditions characterized by pain. As a first-step towards clinical development of amiloride nasal spray, we are conducting an early Phase I clinical study of amiloride nasal spray to evaluate the safety and pharmacokinetics in healthy human volunteers [19].

**Fig 1:**
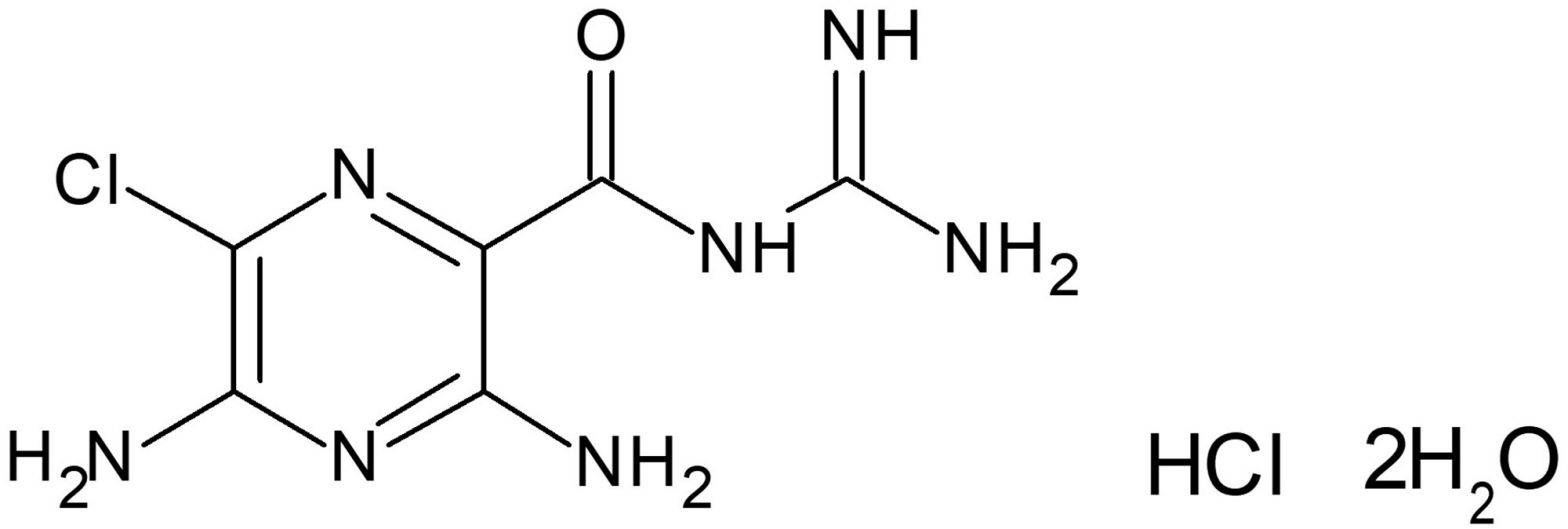
Chemical structure of amiloride hydrochloride dihydrate.

Amiloride hydrochloride is a pyrazine-carbonyl-guanidine [20, 21] salt of a moderately strong base (pKa 8.7), and an antikaliuretic-diuretic agent (Fig 1). Amiloride is currently approved by the Food and Drugs Administration (FDA) as adjunctive treatment with thiazide diuretics or other kaliuretic diuretic agents in congestive heart failure, or hypertension [20]. Amiloride protected the airways from intraluminal obstruction, and improved airflow after inhalation in cystic fibrosis (CF) patients [16]. The efficacy of inhaled amiloride in CF patients was assessed by nasal potential difference measurements and results showed that the duration of inhibition of nasal potential difference was dose dependent [15]. Amiloride is available only as a tablet formulation for oral administration with an onset of action time of 2 hours, with peak plasma levels reached within 3 to 4 hours [20, 21]. This time to onset of action is too slow for panic attacks, which have a rapid onset. However, rapid onset of action can be achieved via the intranasal route of administration that allows for rapid absorption of drugs directly into the brain via the “nose-to-brain” route [22, 23]. The “nose-to-brain” route allows rapid transport of amiloride to brain by circumventing the blood-brain barrier (BBB) [22, 23]. Intranasal administration also provides a non-invasive point of access into the CNS and reduces the risk of needle-stick injuries due to parenteral administration in hospital and emergency department settings [24]. Furthermore, intranasal administration allows for simple, self-administration, which facilitates patients’ adherence [25]. FDA recently (2019) approved Spravato^®^ (esketamine) nasal spray for the treatment of depression, which highlights the importance of the intranasal route of administration for CNS disorders [26].

Because amiloride is an FDA-approved drug, we can extemporaneously compound amiloride nasal spray for the treatment of AD. Amiloride solution was shown to undergo degradation by hydrolysis and photodegradation pathways [27, 28]. Therefore, it is important to establish the stability of amiloride solution for nasal administration. However, currently, there are no reports on the formulation and stability of amiloride nasal spray that can be adapted by the compounding pharmacists to prepare amiloride nasal spray. Therefore, the objective of this study is to develop amiloride nasal spray formulation and report its stability at room temperature. In this study, we report on the extemporaneous formulation of amiloride nasal spray and its physical, chemical, and microbiological stability.

## Materials and methods

### Materials

Amiloride hydrochloride powder (monohydrochloride, dihydrate form) was purchased from EMD Millipore Corporation, Temecula, CA. Lot. 3224630. The United States Pharmacopeia (USP) reference standard of amiloride hydrochloride was purchased from, USP Convention, Rockville, MD. Lot. R052W0. The USP reference standard was used for the HPLC method development and validation. The USP grade sterile water for injection used for the preparation of amiloride nasal spray was purchased from RMBIO, Missoula, MT, USA. HPLC grade glacial acetic acid and methanol were purchased from EMD Millipore Corporation, Temecula, CA, USA. The chemicals and reagents used for the forced degradation studies were purchased from Millipore Sigma, St. Louis, MO, USA. The reversed-phase C18 HPLC column used was Waters Nova-Pak^®^ purchased from Waters Corporation, Milford, MA. Lot. 1130380642.

### Extemporaneous preparation of amiloride nasal spray

Amiloride nasal spray was formulated by dissolving 20 mg of amiloride hydrochloride (monohydrochloride dihydrate form equivalent to 15.2 grams of free amiloride) in 10 mL sterile water for injection (2 mg/mL) in a laminar flow hood. The sterile water for injection was previously heated to 40°C using a water bath. Amiloride hydrochloride exhibits temperature dependent solubility in water [29] and heating water to 40°C before adding amiloride is required to achieve a solubility of 2 mg/mL for amiloride in water. Whenever possible, amiloride was protected from light to avoid photodegradation [27, 30]. The formulation was filtered using 0.22 μm nylon syringe filters into 10-mL sterile syringes in a laminar-airflow hood, Labconco, Logic^+^ A2, Kansas City, MO. 10 mL syringes were chosen as they allow nasal administration of amiloride solution to patients using a MAD Nasal^™^ intransal atomization device [24]. Details of the steps involved in the procedure are provided as S1 Appendix in the supporting information.

### Stability-indicating high-performance liquid chromatography method

The stability-indicating high-performance liquid chromatography (HPLC) method used to analyze amiloride and its degradation products was developed and validated according to FDA guidelines for analytical procedures and method validation for drugs and biologics [31]. Briefly, the mobile phase consisted of a 25:75 ratio of methanol: water with the final pH adjusted to 3.6 with glacial acetic acid. Amiloride was detected using a photodiode array detector at a maximum wavelength of 284 nm. The chromatographic separation was achieved using a 5-μm particle size, 3.9 mm × 15 cm L1 column. The runs for each injection involved an isocratic elution with an equilibration step between each injection programmed, as shown in Table 1.

**Table 1:** HPLC flow program for analysis of amiloride.

Time (min)	Flow rate (mL/min)	% Water	% Methanol
**0.00**	1	75	25
**21.00**	1	75	25
**22.09**	1	100	-
**26.10**	1	100	-
**27.99**	1	75	25

HPLC analysis was performed by injecting 20 μL of the amiloride sample into the separation module equipped with a photodiode array detector, PDA detector, Model No.2998, Waters Corporation, Milford, MA. Data acquisition and analysis were performed using Empower; version 3 (Waters Corp., Milford, MA). The HPLC method was validated according to the International Council on Harmonisation (ICH) guidelines for linearity, accuracy and precision, robustness, and ruggedness [32]. A standard 5-point calibration curve was constructed by linear regression of the peak areas of the amiloride peak obtained from amiloride hydrochloride USP reference standard solutions at concentrations 12.5, 25, 50, 100, and 200 μg/mL (*r*^*2*^ = 0.9998).

The specificity of the HPLC method to degradation products of amiloride was assessed by subjecting amiloride to forced-degradation conditions including hydrolysis, oxidation, photodegradation, thermal degradation, and ultraviolet (UV) irradiation. Stability indicating forced-degradation studies involved treatment of 100 μg/mL amiloride hydrochloride solution with 0.1M hydrochloric acid for 1 hour to assess for acid hydrolysis, 0.1M sodium hydroxide for 1 hour to evaluate for alkaline hydrolysis, 0.1M hydrogen peroxide overnight to determine for oxidation, UV radiation (UV light of laminar-airflow hood) overnight to assess for photostability, and temperature of 60°C (using a laboratory hot plate) overnight to determine for thermal stability studies.

### Chemical stability studies

For the chemical stability analysis, a batch of 20 amiloride nasal spray (2 mg/mL) solutions packed in syringes were prepared as described earlier. Five syringes were randomly selected from the batch and placed on a dry ventilated surface (mean ± S.D. temperature of 22.7 ± 0.8 °C and relative humidity [RH] of 32.5% ± 5%). At 0 (immediately after preparation), 3 hours, 24 hours, and 7 days, a pipette was used to transfer a 100-μL sample of nasal spray into 15 mL centrifuge tube. The solution was diluted to obtain a final concentration of 200 μg/mL. The final solution was injected into HPLC in triplicate for analysis. The percentage assay values for stability samples were calculated using the calibration curve described above, using peak areas of amiloride obtained after integrating peaks from chromatograms of stability samples. The 7 day time-point for stability study was chosen because we did not add any antimicrobial preservatives to our formulation to avoid potential toxicity to the nasal mucosa [33, 34].

### Physical stability tests

Color, visual clarity, pH, and viscosity were also evaluated at 0, 3 hours, 24 hours, and 7 days. The samples were visually inspected against black and white backgrounds using a high-intensity lamp at each time point to evaluate the characteristics of color and clarity. The pH meter, Seven Easy, Model No. S20, Mettler Toledo, Columbus, OH, was calibrated with standard buffer solutions of pH 4, 7, and 10, was used for pH analysis. Viscosity was measured using a rheometer, Kinexus ultra^+^ rheometer, Malvern PANalytical, Malvern, UK.

### Microbiological stability analysis

For microbiological stability analysis, 20 syringes containing amiloride nasal spray solution (2 mg/mL), prepared as described above, were placed on a ventilated surface at room temperature (median ± S.D. temperature of 22.7 ± 0.8 °C and RH of 32.5% ± 5%). Microbiological stability was evaluated at 0 and 7 days after storage at room temperature. To ascertain microbiological stability, samples were subjected to sterility tests described in USP Chapter 71 [35]. The sterility test was carried out using the membrane filtration technique with appropriate negative controls. Briefly, the sample was hand filtered across two separate filters, followed by the addition of tryptic soy broth medium (TSB) to one filter and fluid thioglycollate medium (FTM) to the other. The filter with TSB medium was incubated at 20–25°C and the filter with FTM was incubated at 30–35 °C for over 2 weeks. During these 2 weeks, the media were examined for macroscopic evidence of microbiological growth. Data are presented as the presence or absence of microbial growth as determined by visual examination. All microbiological analyses were performed at Compounder’s International Analytical Laboratory, Castle Rock, Colorado, USA.

### Data analysis

The stability was defined as the retention of at least 90% of the initial concentration of amiloride nasal spray. The chemical stability experiments were performed in triplicate after 5 samples were randomly collected from 20 nasal sprays. Data were represented as the mean ± S.D. percent of the initial concentrations remaining. For pH, clarity, and viscosity tests, differences between samples at different time points were compared using 1-way analysis of variance (ANOVA). The *a priori* level of significance was 0.05. Statistical analyses were performed using GraphPad Prism, Version 8, GraphPad Software, San Diego, CA. For sterility testing, three samples from each time point (0 and 7 days) were filtered in triplicate for each medium.

## Results and discussion

**Fig 2:**
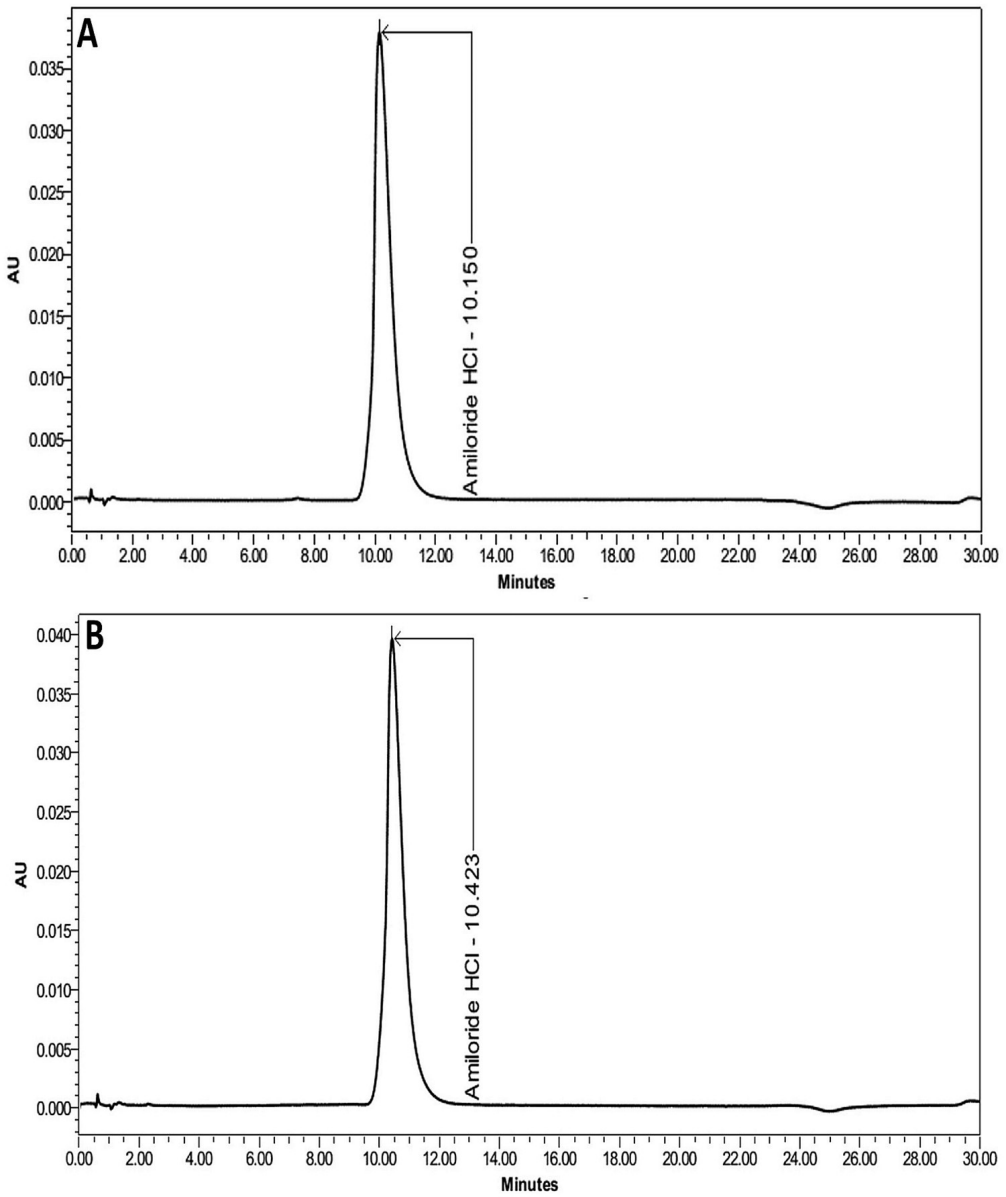
Amiloride hydrochloride pure drug and reference standard chromatograms. Chromatograms representing 0.1 mg/mL amiloride hydrochloride solutions in sterile water for injection. A) 0.1 mg/mL amiloride hydrochloride USP reference standard solution. B) 0.1 mg/mL amiloride hydrochloride solution used for the preparation of nasal spray.

The amiloride nasal spray solutions were successfully prepared and were colorless with no visible particulate matter when inspected against dark and light backgrounds. The validated HPLC method showed that amiloride was eluted at ~ 10.3 minutes. A freshly prepared solution of amiloride hydrochloride was analyzed using the HPLC method and compared with the USP reference standard of amiloride hydrochloride. The retention time and the shape of the peaks were similar between the sample and the standard solutions. The mean ± S.D. percentage assay of amiloride hydrochloride was 96.8% ± 0.4%. The chromatograms showed sharp and distinct peaks for each analyte without interference or co-elution from the contents of the mobile phase (Fig 2).

Relative S.D. for replicate injections was 0.4% (USP limit of ≤2%), indicating the suitability of the HPLC method for the assay of amiloride nasal spray. The standard curve of amiloride nasal spray was linear over the range of concentrations (12.5 to 200 μg/mL, *r*^*2*^ = 0.999). Results from the validation studies showed that the parameters accuracy, precision, robustness, and ruggedness were within the specified limits outlined in FDA guidance for analytical procedures and method validation for drugs and biologics (S2 Appendix) [31].

**Fig 3:**
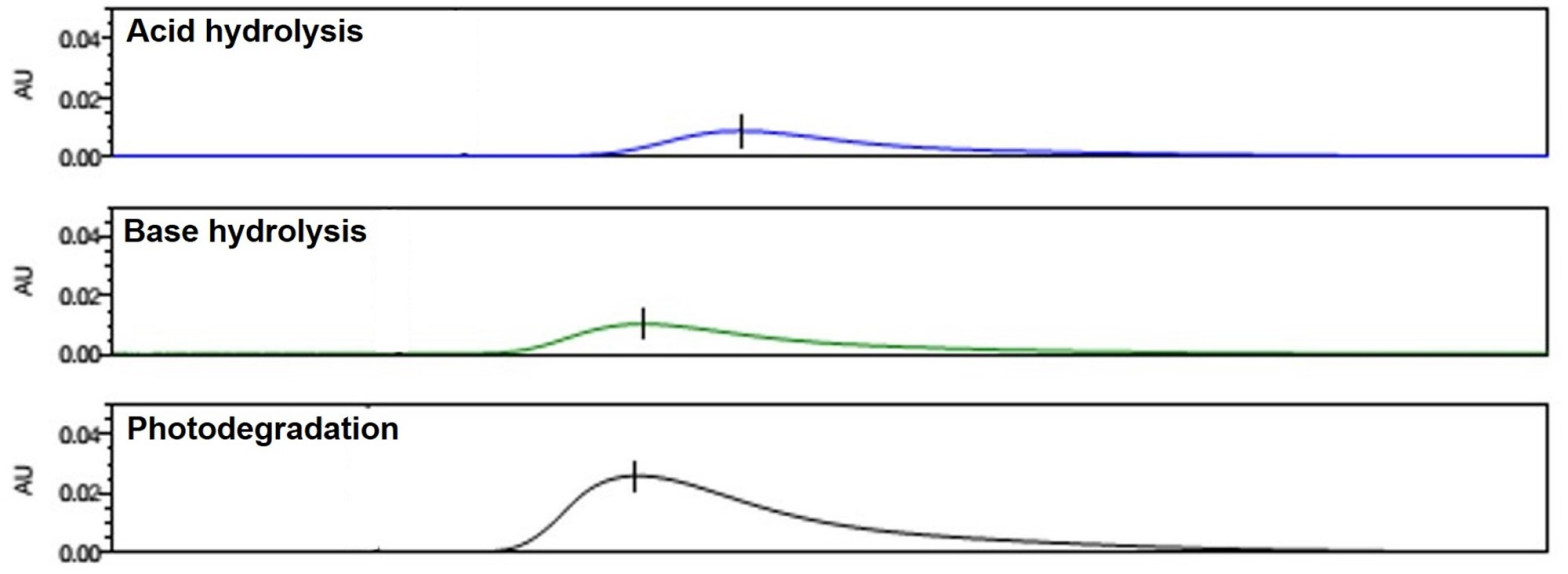
Chromatograms representing forced degradation studies of amiloride. Chromatograms represent from top to bottom represent acid hydrolysis (with HCl), base hydrolysis (with NaoH), and photodegradation.

Hydrolysis and photodegradation are the major degradation pathways of amiloride [27, 28]. Therefore, it is essential that the HPLC method can identify the degradation products of amiloride. The forced degradation studies were performed to assess the capability of the HPLC method to identify the degradation products from intact amiloride distinctly. The chromatograms of amiloride samples subjected to hydrolysis and photodegradation identified the degradation products of amiloride (Fig 3). These results indicate that the HPLC method used has the potential to identify major degradation products of amiloride, and can be successfully used for stability studies of amiloride nasal spray.

**Fig 4:**
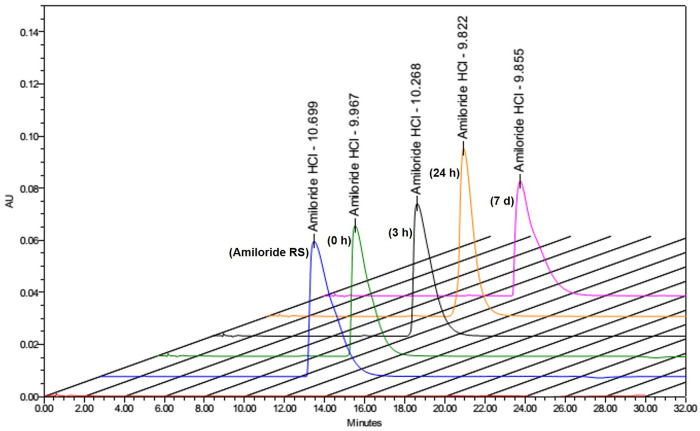
Chemical stability of amiloride nasal spray. Overlay of chromatograms representing amiloride nasal spray samples assayed after storage in bottles at room temperature. From left to right, chromatograms represent USP reference standard, 0, 3 hrs, 24 hrs, and 7 days samples of 0.2 mg/mL amiloride hydrochloride solutions. Amiloride RS–Amiloride USP reference standard.

The amiloride 2-mg/mL nasal spray stored at room temperature showed good physical and chemical stability for up to 7 days (Table 2). The percentage of the initial amiloride concentration remaining at 7 days was 98.7%, indicating satisfactory chemical stability of amiloride nasal spray (Fig 4). However, due to its potential for photodegradation, caution must be exercised by compounding pharmacists to avoid exposing amiloride to light. Furthermore, the nasal spray must be dispensed in containers that preserve amiloride by protection from photodegradation. The data from the physical stability studies (pH and viscosity) showed no significant differences among the samples at time points 0, 3 hours, 24 hours, and 7 days (*p* > 0.05).

**Table 2:** Stability of 2 mg/mL amiloride nasal spray solution.

Stability Parameter Analyzed	0	3 hours	24 hours	7 days
**% Assay of amiloride**^a^	96.5 ± 0.3	96.6 ± 0.2	95.2 ± 0.1	98.2 ± 0.4
**pH**^b^	4.5 ± 0.2	4.5 ± 0.2	4.5 ± 0.3	4.5 ± 0.3
**Viscosity (cP)**^c^	0.9 ± 0.04	0.9 ± 0.02	0.9 ± 0.01	0.9 ± 0.01

^a^ Values represent mean ± S.D. after triplicate analysis of three samples obtained from three nasal sprays. The % assay values are calculated from a calibrated curve prepared from a standard solution of USP amiloride hydrochloride.

^b^Values represent mean ± S.D. pH after triplicate analysis from three nasal sprays. The pH reported is the final pH of the nasal spray after addition of amiloride hydrochloride solution to water. No significant difference in pH was observed between samples at various time points based on results from ANOVA (*p* > 0.05).

^c^Values represent mean ± S.D. after triplicate analysis from three nasal sprays. No significant difference in viscosity was observed between samples at various time points based on results from ANOVA (*p* > 0.05).

FDA requires that all nasal spray solutions for human use must be free of microbiological contamination due to their direct interaction with mucosal surfaces [36]. Therefore, we evaluated the microbiological stability of amiloride nasal spray by confirming its sterility after storage at room temperature for 7 days using the method outlined in USP 71. The results from this testing revealed that the extemporaneously prepared amiloride nasal spray was microbiologically stable for up to 7 days. In all samples, tested, there were no signs of microbiological growth over two weeks.

## Conclusions

An extemporaneously prepared nasal spray solution of amiloride hydrochloride 2 mg/mL exhibited physical, chemical, and microbiological stability over 7 days when stored at room temperature in sterile syringes.

## Supporting information

S1 Appendix Formulation of amiloride nasal spray. Document with step-by-step directions for extemporaneously compounding amiloride nasal spray 2 mg/mL.
(DOCX)

S2 Appendix HPLC method validation results. Excel file containing HPLC method validation data for amiloride hydrochloride.
(XLSX)
