# Supplementary material for: Stability of extemporaneously compounded amiloride nasal spray
Source: PLoS One. 2020 Jul 10;15(7):e0232435. doi: 10.1371/journal.pone.0232435 (PMC7351165; doi:10.1371/journal.pone.0232435)
Supplement: S1 Appendix — Document with step-by-step directions for extemporaneously compounding amiloride nasal spray 2 mg/mL. (DOCX) [file pone.0232435.s001.docx]

**S1. Appendix – Procedure for compounding 2-mg/mL amiloride nasal spray**

1. In a laminar flow hood, add 100 mg of amiloride Hydrochloride powder and 50 mL sterile water for injection in a sterile 100 mL amber colored reagent bottle and close the lid tightly.
2. Transfer the bottle to a water bath preheated to 35°C and leave it for one hour or until the amiloride dissolves completely. Gently shake the bottle to ensure amiloride is dissolved.
3. Filter the amiloride solution using 0.22-µm nylon syringe filters in a laminar airflow hood and fill sterile syringes with the amiloride solution and cover them with aluminous foil to protect from light until further use.
4. Attach MAD Nasal^TM^ intranasal atomization device to the syringe and administer to patients intranasally.
